# Unhygienic Practices of Health Professionals in Brazilian Public Hospital Restaurants: An Alert to Promote New Policies and Hygiene Practices in the Hospitals

**DOI:** 10.3390/ijerph16071224

**Published:** 2019-04-05

**Authors:** Cainara Lins Draeger, Rita de Cassia Coelho de Almeida Akutsu, Karin Eleonora Sávio de Oliveira, Izabel Cristina Rodrigues da Silva, Raquel Braz Assunção Botelho, Renata Puppin Zandonadi

**Affiliations:** 1 Department of Nutrition, Faculty of Health Sciences, University of Brasilia, Brasilia 70910-900, Brazil; cainara@gmail.com (C.L.D.); rita.akutsu@gmail.com (R.d.C.C.d.A.A.); kesavio@unb.br (K.E.S.d.O.); raquelbabotelho@gmail.com (R.B.A.B.); 2 Faculty of Ceilândia, University of Brasília, Brasília 72220-275, Brazil; belbiomedica@gmail.com

**Keywords:** contamination, prevention, health professionals, hospital restaurant, unhygienic practices

## Abstract

This study aimed to evaluate health professionals’ unhygienic practices and the stages of behaviour change in Brazilian public hospital restaurants. We evaluated all medium/large-sized public hospital restaurants (HRs) from the Brazilian Federal District (*n* = 9); a representative sample of 128 users). We evaluated the HRs’ physical structures, their consumers’ socio-demographic characteristics, their unhygienic practices, as well as the stages of behavioural change concerning unhygienic practices. All the HRs presented their menus for self-service distribution, so customers entered in lines to serve themselves. All the HRs had hand-wash sinks for customers; 77.8% offered antiseptic liquid soap; 33.3% offered alcohol gel; and 77.8% offered storage for professional accessories before serving food. Almost half (46.8%) of the customers did not sanitise their hands (with water and antiseptic soap and/or use of alcohol gel) immediately before serving, and 24.2% wore professional uniforms at HRs. Almost half (43.5%) of the customers spoke with each other in line while serving their plates and arranged the food on their plates with the serving utensils from the distribution counter. The declaration of behavioural change was inversely associated with the hygiene practices. Almost half of the individuals did not sanitize their hands; however, 90.4% declared “changed behaviour” when this contaminant practice was presented to them. We verified a high percentage of hygiene practices inconsistent with most of the customers´ answers about their stage of behaviour change. Based on the observations of this study, it is necessary for an awareness program to be developed that is focused on customers of HRs in order to reduce unhygienic practices. Also, it is important to promote new policies for proper hygiene practices in hospital restaurants.

## 1. Introduction

Different from their expectations (in which the cook is usually blamed), customers can be a source of food contamination (FC). FC can occur in many parts of the production and supply of food, including during food consumption when customers have direct contact with the meal [1].

In hospitals, health professionals are potential hospital restaurant (HR) customers. They should be aware of the possibility of food contamination by themselves, as they are in contact with sick patients, who may be transmitters of several pathogens. Contamination of the skin and clothing by splashing and touching is virtually inevitable. Although the hands of healthcare personnel most commonly transmit microorganisms, materials and articles used in the hospitals could also carry microorganisms. It is known that accessories used by hospital professionals can be a potential source of infections. Therefore, working clothes, articles, and utensils may be considered an important route of infection transmission [2,3,4,5]. The physical structure of the hospital restaurants can minimise contamination within the hospital settings, and thus there should be proper planning to prevent contamination and to ensure consumers´ safety and satisfaction [6,7]. There are regulations in Brazil regarding the physical structure of restaurants, as well as regulations on good production practices that should be followed when preparing food. It is mandatory to follow these regulations, and the government presents professionals for official control in different types of restaurants, including inside hospitals. Besides the regulations and proper structure of the restaurants, knowledge about proper hygiene practices, the availability of hygiene resources, and the awareness of hospital restaurants’ customers about food hygiene practices can be considered essential tools in preventing foodborne diseases (FbDs). Usually, customers do not understand their responsibility to prevent FbDs, as they do not see themselves as potential hazards. They need to be aware and to understand that some habits must be changed when entering a restaurant. We still lack studies that evaluate adequate hygiene practices from customers and the availability of hygiene resources for this population, or studies about the awareness of customers from hospital restaurants that they can be important sources of contamination for food preparations.

In addition to assessing hygiene hospital professional practices in HRs, it is essential to evaluate the stages of behaviour change regarding this problem. Changing habits and behaviour is a gradual process, and it involves many factors. The transtheoretical model can be used to assist the planning of educational actions in the search for appropriate behaviours. This model allows the stage of behaviour change of a studied group to be identified. The stages are as follows: pre-contemplation (the behaviour change has not yet been considered, and there is no intention of adopting it in the near future), contemplation (individuals begin to consider the change and intend to change in the future without a definite term), decision (individuals intend to change in the near future, within the next 30 days), action (individuals have already changed their behaviour, and such changes are visible and recent, as within the last six months) or maintenance (individuals have already modified their behaviour and maintained it for more than six months) [8]. The identification of the stages of change allows strategies to be proposed that support new approaches to behaviour change and the adoption of healthy behaviours for interventions in several areas such as psychology, medicine, and nutrition [9,10]. Therefore, this study aimed to evaluate professional hygiene practices and the stages of behaviour change in Brazilian public hospital restaurants. This knowledge will be healpful to show health professionals how their practices inside hospital restaurants can impact their health.

## 2. Materials and Methods

### 2.1. Research Outline

This is an exploratory cross-sectional study. Sampling comprised all restaurants from public hospitals located in the Federal District, Brazil (*n* = 9), classified as medium and large-sized restaurants (above 100 meals per day) [11]. Customers (hospital professionals) who had lunch at the HRs composed the study population sample (average of 288 ± 118 customers per unit). We considered a confidence level of 95% to calculate the sample, with a margin of error of 5%, and a population size of 1500 with a response of distribution of 5%. The result was a minimum initial sample of 120 customers distributed according to the number of customers of each of the nine HRs. Only health professional working 12-h or 24-h shifts could have lunch at the hospital restaurants. Administrative workers with the same working shifts were the ones in the surgical units or milk dispensary areas.

We observed the customers’ hygiene practices by systematic sampling: one consumer was selected out of every 20 who entered the restaurant. After the observation of the hygiene practices of each customer while serving the meal, we explained the research, and we asked each costumer if we could use the data, and then we invited the costumer to participate in the next steps. We chose this strategy of selecting one out of 20 customers as a way of assuring the homogeneity of the sample [12].

### 2.2. Stages of Data Collection

Data collection was carried out for one day in each HR, and it was divided into three steps.

In Step 1, before the opening of the restaurant to customers, the researchers checked the hygiene equipment and supplies: the sink(s) near the entrance or inside the restaurant, the type of water activation (automatic or manual), alcohol gel, antiseptic soap, non-recycled paper towel and self-storage (Supplementary File Table S1), as recommended by the World Health Organization (WHO) [13].

In Step 2, the Hygiene Practices instrument was used (Supplementary Materials, Tables S2 and S3). It was developed based on the study of Zandonadi et al. [1], which recorded the number of users who entered the restaurant wearing working uniforms (coats, surgical scrubs, and uniforms worn by the hospital laundry staff) and/or carrying working tools such as stethoscopes, thermometers, sphygmomanometers, and suitcases containing such objects. In this step, personal hygiene aspects related to the HR customers were considered (hand hygiene by washing hands with water and antiseptic soap and/or use of alcohol gel immediately before serving or after contact with the patients). Also, hygiene practices were observed when customers were serving food at the counter (users who talked; touched their hair; rubbed their noses and/or eyes; returned the food already served to the food display modules; organized the food on the plate with the serving utensils). All of these actions were considered unhygienic practices. In this step, customers were observed while serving without knowing that their actions were evaluated. This was important to avoid any influence over the behaviour of the participants.

In Step 3, we created and applied the Stages of Change instrument that was based on the studies of Toral and Slater [9] and Zandonadi et al. [1] (Supplementary Materials Table S4). This instrument contained closed questions and it was filled out by the customers themselves. The objective of the instrument was to identify the socio-demographic characteristics and the stages of behaviour change regarding hygiene practices observed at HRs. The forms of information collected by the instrument were gender, educational level, employment position, working sector, and age, as well as the stage of behaviour changes in a five-point Likert scale, ranging from 1 (Precontemplation) to 5 (Maintenance).

### 2.3. Statistical Analysis

We described the measures of central tendency and variability of the sample. Pearson’s chi-square test and Spearman’s rank-order correlation were used to analyse the results (the results were considered significant if *p* < 0.05). We applied a linear regression model to verify the unhygienic practices. The independent variables were the ones related to practices while serving food (serving without hand washing; speaking while serving; returning the food already served; and organizing the food on the plate with the serving utensils). The dependent variable was the contamination behaviour (based on the dichotomised score). This variable was created with the following condition: if the participant of the research was identified as having displayed some form of inadequate hygiene practice or the structure of the place did not have an instrument for hygiene, code 0 (i.e., code 0 = contaminant practice) was entered, otherwise code 1 was assigned. The objective was to determine which practices most impacted the study. The model was estimated with all variables; at the end of the estimation, those with statistical significance less than 5% (*p* < 0.05) were selected. Collinearity was evaluated by the variance inflation factor (IVF), in which values lower than 10 indicate the absence of collinearity problems. No variables were found that did not meet this requirement. Then, the final model for the equation was based on the variables with significance.

A dichotomised score was used to perform the Stages of Change analysis. From the Likert scale, a score of 1 was classified as Precontemplation, with answers ranging from “I don’t think about it or don’t do that”. A score of 2 was classified as Contemplation, with answers like “I’ve been thinking this way”. A score of 3 was classified as Decision, with a statement such as “I’m determined to do that”. A score of 4 was classified as Action (“I started doing it recently”), and a score of 5 was classified as Maintenance (“I have done this for a long time”). We divided the stages into Action and Maintenance (Changed Behaviour, scores 4 and 5) and Precontemplation, Contemplation, and Decision (No Behaviour Change, scores 1, 2 and 3). We used SPSS^®^ version 22.0 (IBM, Armonk, NY, USA) for the statistical analysis.

### 2.4. Ethical Aspects

The Ethics Committee FapDF from Federal District, Brazil (n.065/2012) approved this project. The research was carried out with the customers who agreed to participate by signing an informed consent form (ICF). 

## 3. Results

All the HRs presented sinks for hand-washing of which 55.6% (*n* = 5) of them had rotary activation faucet sinks (manual activation), and the others had sinks with pressure activation faucets (manual activation). Almost 80% (*n* = 7) of the HRs offered antiseptic liquid soap, and 33% (*n* = 3) offered alcohol gel. All the HRs provided non-recycled paper towels and 77.8% (*n* = 7) of them provided self-storage (shelves, lockers, racks or tables) for customers to leave their belongings before serving the food.

The instruments of Steps 2 and 3 were applied to 128 customers; four did not respond to the questionnaire provided. The final sample was composed of 124 customers with a mean age of 41 ± 11.1 years, and 58.1% (*n* = 72) were female. Table 1 summarises the socio-demographic data.

The results related to the Step 2 instrument show that 46.8% (*n* = 58) of the customers did not perform hand hygiene (washing hands with water and antiseptic soap and/or use of alcohol gel), 24.2% (*n* = 30) entered the restaurant wearing their working uniforms, and 0.8% (*n* = 1) carried a working instrument to the restaurant. It was verified that in hospital restaurants where alcohol gel was offered, none of the customers used it. No significant correlations were found between the socio-demographic data and hand hygiene (washing hands with water and antiseptic soap); use of alcohol gel; serving while wearing a work uniform; and serving food with working instruments.

We analysed the relationship between the results of Step 2 (Hygiene Practices) and Step 1 (records about the presence of hygiene resources: equipment and supplies). The association between hand hygiene (washing hands with water and antiseptic soap) and the type of soap (antiseptic and common soap) provided by the HRs was significant. When the hospital restaurant provided antiseptic soap, there was greater number of customers who cleaned their hands (*χ*^2^ = 13.802; df = 1; *p* < 0.001; ρ = 0.334). The presence of a self-storage area at the hospital restaurants contributed to a lower use of working uniforms in HRs (*χ*^2^ = 9.207; df = 1; *p* = 0.002; ρ = −0.272).

Table 2 shows the correlation between the results of Step 2 (Unhygienic Practices) and Step 3 (Stages of Change—Changed Behaviour, represented by the Action and the Maintenance stages). The overall proportion of individuals who declared changed behaviour was inversely related to the number of individuals who were observed performing unhygienic practices. It is noteworthy that while almost half of the individuals did not clean their hands (washing hands with water and antiseptic soap and/or use of alcohol gel), virtually the entire study sample (90.4%) declared changed behaviour concerning the unhygienic practices studied.

In order to determine which items of the contamination practice (independent variables) instrument most influenced the contamination behaviour (dependent variable), we performed a linear regression model analysis. Thus, the equation proposed for the model related to the presence of a contamination practice is as follows:

(*p* (*contamination practice*)/1 − *p* (*contamination practice*)) = 1.62*x*_1_ + 1.42*x*_2_ + 1.26*x*_3_ + 0.98*x*_4_

where *x*_1_ represents serving food without washing/sanitizing hands; *x*_2_ represents speaking while serving; *x*_3_ represents returning food already served; and *x*_4_ represents touching the food on the plate with serving utensils. 

According to the linear regression model, serving food without washing hands was the practice that most influenced the unhygienic practices followed by talking while serving.

## 4. Discussion

This is the first study to discuss the relationship among the structure, products, and services of HRs, unhygienic practices adopted by customers and the stages of behavioural change in restaurants of public hospitals. We observed that the same HRs that did not offer antiseptic soaps did not offer alcohol gel for hand sanitisation, which is considered inappropriate according to the World Health Organization [13]. It is important to highlight that in the absence of antiseptic soap, alcohol gel should be offered for proper hand sanitisation with common soap. Health services should provide antiseptic soaps so that the hospital professionals can sanitise their hands. 

The lack of hand hygiene within health services is related to the spread of microorganisms from patients to restaurants utensils, then to other health professionals that will serve food afterwards. Health professionals can spread microorganisms to different patients by serving food with contaminated utensils [5]. This is directly related to increases in mortality and morbidity, length of hospitalisation, and an increase in financial expenses associated with the treatment of patients [14,15].

An adequate physical structure of hospital restaurants, as well as the availability of equipment (sinks, bins, and soap and paper towel dispensers), input (water) and supplies (paper towels, antiseptic soaps, and alcoholic preparations), favours the prevention of any form of contamination within a hospital setting [6]. According to Anderson et al. [7], the physical structures of HRs should be designed to facilitate procedures that prevent contamination, and they should ensure that users are satisfied with the resources available. Ideally, the available hygiene equipment and supplies should call the attention of the customer (for example, put stickers on the alcohol gel dispenser with an eye-catching message for their use). This strategy aims to encourage customers to use available hygiene equipment and supplies and to adopt proper hygiene practices.

According to the WHO, hospitals must provide equipment, devices, and products that facilitate hand hygiene. Hospitals, as well as their restaurants, must have hands-free faucets on their sinks that do not require manual contact. Alternatively, when contact is unavoidable, non-recycled paper towels should be used for turning off water taps. Besides that, the WHO notes that the inadequate location (out of circulation or service) of hand sanitisation dispensers and absence of hand washing with soap or alcohol gel contribute to the poor adherence to hand hygiene by health professionals. Furthermore, the Guidelines for Design and Construction of Health Care Facilities of the American Society of Healthcare Engineering of the American Hospital Association also addresses the need for providing basic hygiene supplies in hospital settings [16,17].

In Brazil, according to the Brazilian legal Resolution No. 50 issued on 21 February 2002, health services must have washbasins/sinks/stalls with faucets or another type of hands-free control. Also, a degerming liquid soap must be provided, as well as hand-drying supplies or devices [18]. Therefore, besides putting the health of their patients at risk, the practices of the evaluated hospitals are in disagreement with the international rules and regulations as well as with the Brazilian sanitary legislation. 

Socio-demographic data (Table 1) show that most of the HRs’ customers had completed higher education and were the health professionals who work at the hospitals. As such, we can infer that most of the HRs’ customers were aware of proper hygiene practices to prevent food contamination and foodborne diseases. The hygiene practices information is widely available at national and international levels and used for health professional training [18,19].

The low adherence to hand hygiene found in this study is in line with the findings of other studies [20,21], and it shows a critical situation within hospitals, since hand hygiene is considered a primary measure of hospital infection control and the prevention of foodborne diseases. The practice of hand hygiene reduces the transmission of microorganisms significantly and, consequently, it reduces infections as well as the morbidity and mortality rates in health services [5,6,22,23]. Many health professionals were wearing their working uniforms in HRs. Other studies also mention the use of working uniforms in other environments, such as public places, snack bars, supermarkets, and on public transportation [24,25]. This practice indicates that health professionals are not aware of the risk of wearing their uniforms outside their work environment. These uniforms are considered vehicles of microorganisms’ dissemination [3,25].

From the analysis of the stages of behavioural change instrument, we observed that customers were in the maintenance stage, which means that they have recognised their change in behaviour and its maintenance for more than six months, and that such behaviour has been consolidated and incorporated into their routine [8,9]. We believe that customers of this study may be in the contemplation stage rather than the maintenance stage, as they have classified themselves. This can be justified by the fact that in the contemplation stage there is knowledge of the benefits of change, but several barriers are preventing the individual(s) from performing the desired action [9,26]. It is important to mention that the health professionals did not recognise themselves as potential contaminators [5]. Most of the users have an education background in which knowledge about hygiene is part of their curriculum; this fact demonstrates a significant discrepancy between their discourse and their practice. Thus, customers admit knowing the appropriate practices when answering the stages of behavioural change instrument, but for some reason they do not adopt the practices in their everyday lives.

According to our results, there is a contradiction between the observed practices and the answers provided by HR customers. The most worrisome finding concerns hand hygiene before serving. Hand hygiene is considered the most critical measure for the prevention of foodborne diseases and the reduction of intra-hospital infections. Even though it is a simple, and inexpensive procedure, health professionals still do not do it properly, which is a problem that has been observed throughout the world [27].

According to Primo et al. [28], the low habit of hand hygiene among customers (mostly health professionals) is not directly associated with their theoretical knowledge about hand hygiene, but rather with the application of this knowledge to their daily lives and professional habits. Different reasons are related to the health professionals’ negligence about the behaviour of hand hygiene to prevent diseases and infections: personal beliefs, myths, lack of adequate physical structure, lack of motivation, time, qualifications, and awareness about the significance of their behaviour to the transmission of microorganisms [29,30].

Thus, the transtheoretical model can help with planning educational measures for non-hygienic behaviours. However, we should consider the observed hygiene practices, not the declaration on the behavioural stage change (Stages of Change instrument) for the adoption of educational strategies. The identification of the stage and the analysis of the practices performed are defining aspects for choosing intervention strategies so that the change or the maintenance of the behaviour towards what is desired may take place [9,31].

## 5. Conclusions

Studies on sources of hospital restaurants’ unhygienic practices should be encouraged, as the discovery of new sources of information can provide the development of new approaches for the training and control of hospital infection. Also, this study can enhance discussions about the development of new educational and control strategies that can be implemented in countries experiencing a similar situation. Almost half of the HR customers did not wash their hands prior to entering the restaurant and thus risked contaminating utensils while serving their meals. The actions of HR customers are essential to ensuring their safety (own health) as well as the health of patients, co-workers, and other restaurant users. Knowledge of microbiology and hygiene practices, which are part of health trainings, should be applied for protection in routine activities, including when eating inside hospital areas. In addition to the high percentage of practices performed, the users’ answers about the stages of behavioural change are not in line with the observed unhygienic practices. We recommend the development of an awareness program focused on the users of HRs that is associated with the hygiene equipment and supplies that promote proper hygiene practices (self-storage, sinks, antiseptic soaps, paper towels, and alcohol gel). This study has the following methodological limitations: the lack of observation of the behaviour of the customers during all other operating hours of the HRs (breakfast and dinner); the lack of observation of the correct technique of hand hygiene, since this study only analysed if customers washed hands before serving food or after attending their patient(s); and the nature of the study, as the variables were estimated at a single point of time.

## Figures and Tables

**Table 1:** Socio-demographic data of health professional users of public hospital restaurants (Federal District, Brazil).

Variables		*N*	%
Age (years)	Up to 30	23	18.5%
	31 to 40	45	36.3%
	41 to 50	25	20.2%
	51 above	31	25.0%
Sex	Male	52	41.9%
	Female	72	58.1%
Educational level	Incomplete elementary education	3	2.4%
	Complete elementary education	2	1.6%
	Incomplete high school education	9	7.3%
	Complete high school education	32	25.8%
	Incomplete undergraduate education	17	13.7%
	Undergraduate	61	49.2%
Hospital position	Doctor	9	7.3%
	Nurse	16	12.9%
	Nursing assistant	48	38.7%
	Health equipment operator	5	4.0%
	Administrative assistant	22	17.7%
	Operational assistant of other services	7	5.6%
	Others	17	13.7%
Working department	Clinical unit	29	23.4%
	Surgical unit	7	5.6%
	Emergency unit	20	16.1%
	ICU **/Neonatal ICU	16	12.9%
	Obstetric unit	8	6.5%
	Administration *	15	12.1%
	Mobile emergency care	7	5.6%
	More than one clinical unit	8	6.5%
	Others	14	11.3%

* Administration workers are the ones working inside the surgical units and milk dispensaries. ** ICU—Intensive care unit.

**Table 2:** Correlation between the proportion of unhygienic practices and the stages of behaviour change (Changed Behaviour: represented by the Action and the Maintenance stages).

Practices Performed x Stages of Behavioural Change—Action and Maintenance (Changed Behaviour)	Practices Performed ^#^		Stages of Behavioural Change—Action and Maintenance		*p*
	%	*N*	%	*n*	
Serving the food without washing/sanitizing hands ^◊^	46.8	58	90.4	112	<0.001 *
Wearing working uniform at the restaurants ^$^	24.2	30	67.7	63	<0.001 *
Carrying working tools in the restaurant ^$^	0.8	1	95.6	66	<0.001 *
Speaking, sneezing, coughing while serving	43.5	54	75	93	0.002 *
Eating/testing food while serving	0	0	96	119	0.002 *
Touching hair while serving	11.3	14	82.2	102	0.147
Touching food on the plate with serving utensils	43.5	54	86.3	107	<0.001 *
Returning the food served on the plate back to the main utensil by hand or serving utensils	19.4	24	95.2	118	<0.001 *
Serving the food with own hands	1.6	2	92.8	115	0.032 *

# The participant may have shown more than one unhygienic practice; ^$^ The practice had a smaller number of respondents because the question may not apply to the responder (example: position that does not need to wear uniforms or use working tools); * *p* < 0.05; ^◊^ Lack of washing hands with water and antiseptic soap and/or use of alcohol gel immediately before serving.

## Supplementary Materials

The following are available online at https://www.mdpi.com/1660-4601/16/7/1224/s1, Table S1: Registration of the available resources in the public hospitals of the Brazilian Federal District; Table S2: Unhygienic practices instrument part 1: registration of the use of work uniforms and working instruments, hand hygiene and/or the use of the alcohol gel; Table S3: Unhygienic practices instrument part 2: registration of customers´ contaminating practices during dish assembling; Table S4: Questionnaire to identify the stage of behavioural change in regard to contaminating practices in hospital restaurants.

## References

[1] Zandonadi R.P., Botelho R.B.A., Sávio K.E.O., Akutsu R.D.C., Araújo W.M.C. (2007). Atitudes de risco do consumidor em restaurantes de auto-serviço. Rev. Nutr..

[2] Loveday H.P., Wilson J.A., Hoffman P.N., Pratt R.J. (2007). Public perception and the social and microbiological significance of uniforms in the prevention and control of healthcare-associated infections: An evidence review. Br. J. Infect. Control.

[3] Treakle A.M., Thom K.A., Furuno J.P., Strauss S.M., Harris A.D., Perencevich E.N. (2009). Bacterial contamination of health care workers’ white coats. Am. J. Infect. Control.

[4] Oliveira J.B., Zandonadi: R.P. Effect of Transglutaminase in Baked Products for Celiacs -Review. Nutricao Pauta..

[5] Pandey A., Asthana A., Tiwari R., Kumar L., Das A., Madan M. (2010). Physician accessories: Doctor, what you carry is every patient’s worry?. Indian J. Pathol. Microbiol..

[6] Allegranzi B., Pittet D. (2009). Role of hand hygiene in healthcare-associated infection prevention. J. Hosp. Infect..

[7] Anderson J., Gosbee L.L., Bessesen M., Williams L. (2010). Using human factors engineering to improve the effectiveness of infection prevention and control. Crit. Care Med..

[8] Norcross J.C., Krebs P.M., Prochaska J.O. (2011). Stages of change. J. Clin. Psychol..

[9] Toral N., Slater B. (2007). Abordagem do modelo transteórico no comportamento alimentar. Cienc. Saude Coletiva.

[10] Kosma M., Cardinal B.J. (2016). The Transtheoretical Model, Physical Activity, and Falls Risks Among Diverse Older Adults. Act. Adapt. Aging.

[11] Brasil DOU sectio 1 page *1058.Decreto No 5, de 14 de Janeiro DE 1991*; Brasilia-DF.

[12] Cochran W.G. (2007). Sampling Techniques.

[13] World Health Organization (WHO) World Alliance for Safer Health Care (2009). WHO Guidelines on Hand Hygiene in Health Care. First Global Patient Safety Challenge Clean Care Is Safer Care.

[14] Orsi G.B., Raponi M., Franchi C., Rocco M., Mancini C., Venditti M. (2005). Surveillance and infection control in an intensive care unit. Infect. Control Hosp. Epidemiol..

[15] Leblebicioglu H., Rodriguez-Morales A.J., Rossolini G.M., López-Vélez R., Zahar J.-R., Rello J. (2016). Management of infections in critically ill returning travellers in the intensive care unit—I: Considerations on infection control and transmission of resistance. Int. J. Infect. Dis..

[16] Bartley J.M., Olmsted R.N., Haas J. (2010). Current views of health care design and construction: Practical implications for safer, cleaner environments. Am. J. Infect. Control.

[17] Denholm B. (2010). Guidelines for design and construction of health care facilities. AORN J..

[18] *Brasil* DOU 30 *section1, page 24.RESOLUTION-RDC N° 50, DE 21 DE FEVEREIRO DE 2002* Brasilia-DF.

[19] Scallan E., Hoekstra R.M., Angulo F.J., Tauxe R.V., Widdowson M.-A., Roy S.L., Jones J.L., Griffin P.M. (2011). Foodborne illness acquired in the United States—Major pathogens. Emerg. Infect Dis..

[20] Novoa A.M., Pi-Sunyer T., Sala M., Molins E., Castells X. (2007). Evaluation of hand hygiene adherence in a tertiary hospital. Am. J. Infect. Control.

[21] Schneider J., Moromisato D., Zemetra B., Rizzi-Wagner L., Rivero N., Mason W., Imperial-Perez F., Ross L. (2009). Hand hygiene adherence is influenced by the behaviour of role models. Pediatr. Crit. Care Med..

[22] Loveday H.P., Wilson J., Pratt R.J., Golsorkhi M., Tingle A., Bak A., Browne J., Prieto J., Wilcox M. (2014). epic3: National evidence-based guidelines for preventing healthcare-associated infections in NHS hospitals in England. J. Hosp. Infect..

[23] Medeiros E.A., Grinberg G., Rosenthal V.D., Angelieri D.B., Ferreira I.B., Cechinel R.B., Zanandrea B.B., Rohnkohl C., Regalin M., Spessatto J.L. (2015). Impact of the International Nosocomial Infection Control Consortium (INICC) multidimensional hand hygiene approach in 3 cities in Brazil. Am. J. Infect. Control.

[24] Loh W., Ng V.V., Holton J. (2000). Bacterial flora on the white coats of medical students. J. Hosp. Infect..

[25] Uneke C.J., Ijeoma P.A. (2010). The potential for nosocomial infection transmission by white coats used by physicians in Nigeria: Implications for improved patient-safety initiatives. World Health Popul..

[26] Glanz K., Rimer B.K., Viswanath K. (2008). Health Behaviour and Health Education: Theory, Research, and Practice.

[27] World Health Organization (2007). Food Safety and Foodborne Illness.

[28] Primo M.G.B., Ribeiro L.C.M., da Silva Figueiredo L.F., Sirico S.C.A., de Souza M.A. (2010). Adesão à prática de higienização das mãos por profissionais de saúde de um Hospital Universitário. Rev. Eletrônica Enferm..

[29] O’Boyle C.A., Henly S.J., Larson E. (2001). Understanding adherence to hand hygiene recommendations: The theory of planned behaviour. Am. J. Infect. Control.

[30] Wilson J.A., Loveday H.P., Hoffman P.N., Pratt R.J. (2007). Uniform: An evidence review of the microbiological significance of uniforms and uniform policy in the prevention and control of healthcare-associated infections. Report to the Department of Health (England). J. Hosp. Infect..

[31] Prochaska J.O., DiClemente C.C., Norcross J.C. (1992). In search of how people change. Applications to addictive behaviours. Am. Psychol..

