# Supplementary material for: Unhygienic Practices of Health Professionals in Brazilian Public Hospital Restaurants: An Alert to Promote New Policies and Hygiene Practices in the Hospitals
Source: Int J Environ Res Public Health. 2019 Apr 5;16(7):1224. doi: 10.3390/ijerph16071224 (PMC6479858; doi:10.3390/ijerph16071224)
Supplement: Supplementary file 1 [file ijerph-16-01224-s001.pdf]

Supplementary materials

Table 1. Registration of the available equipment and supplies in the public hospitals of the Brazilian Federal District.

Structure / Hospital	H1	H2	H3	H4	H5	H6	H7	H8	H9
Sink<br>• Manual operation<br>• Automatic operation									
Liquid soap<br>• Antiseptic<br>• Common									
Paper towel<br>• Recycled<br>• Non-recycled									
Alcohol gel									

Table S2. Unhygienic practices instrument part 1: registration of the wearing of work uniforms and working instruments, hand washing and/or the use of alcohol gel.

Consumer	Use of work uniforms (Yes/No)	Use of working instruments (Yes/No)	Hand hygiene with water and soap (Yes/No)	Hand hygiene with alcohol gel (Yes/No)
1				
2				
3				
4				
5				
6				
7				
8				
9				
10				
11				
12				
13				
14				
15				
16				
17				
18				
19				
20				

Table S3. Contamination Practices instrument part 2: registration of consumers´ contaminating practices during dish assembling.

PRACTICES	1	2	3	4	5	6	7	8	9	10	11	12	13	14	15	16	17	18	19	20
Wearing working uniforms when serving																				
Carrying working tools in the restaurant																				
Letting part of the uniform touch the food																				
Speaking, sneezing, coughing while serving																				
Serving food with their hands to their plate																				
Scratching their nose and/or eyes while serving																				
Touching their hair while serving																				
Removing food already served on the plate and returning it to the vats																				
Eating/testing food while serving																				
Touching food on the plate with serving utensils																				
Letting the whole utensil fall into the prepared food																				
Leaning over the plate of prepared food																				

Table S4. Questionnaire to identify the stage of behavioural change in regard to unhygienic practices in hospital restaurants

**Questionnaire to identify the stage of behavioural change in regard to unhygienic practices in hospital restaurants**	
**1) Date of birth?**<br><br>__ __/ __ __/ __ __	
**2) Gender?**<br>1. □ Male<br>2. □ Female	
**3) What is your educational level?**<br>1. ◎ Illiterate<br>2. ◎ Incomplete elementary<br>3. ◎ Complete elementary<br>4. ◎ Incomplete high school<br>5. ◎ Complete high school<br>6. ◎ Incomplete undergraduate<br>7. ◎ Complete undergraduate<br>8. ◎ Special education	
**4) What is your job position?**<br>1. □ Doctor<br>2. □ Nursery<br>3. □ Dentist<br>4. □ Chemist<br>5. □ Nutritionist<br>6. □ Nursing technician<br>7. □ General Service Assistant<br>8. □ Cleaning assistant<br>9. □ Administrative agent<br>10.□ Other: ____________________	
**4) What is your working sector at the hospital?**<br>1. ◎ First aid<br>2. ◎ Maternity<br>3. ◎ Surgical Center<br>4. ◎ Medical Clinic<br>5. ◎ Pediatrics<br>6. ◎ Administration<br>7. ◎ Laundry<br>8. ◎ Intensive care unit<br>9. ◎ Pharmacy<br>10. ◎ Other: ____________________	
For each of the questions below, check the option that best represents your behavior in the following situations:	
**5) Hygiene of the hands before meals.**<br>1. ◎ I do not do it, and I do not feel like doing it.<br>2. ◎ I do not perform it, but I know that I need to change and perform it properly. I intend to do this within six months.<br>3. ◎ I do not perform it, but I know I need to change and perform it properly. I intend to do this within 30 days.<br>4. ◎ I performed adequately and started to perform adequately less than six months ago.<br>5. ◎ I perform properly and have been performing properly for more than six months.	
6) **Use of work clothes at the hospital restaurant**. Note that working clothes are protective clothing used in the work environment, such as lab coats, aprons, caps, hats, masks and so on.<br>1. ◎ I use working clothes and I do not feel like stopping wearing my clothes while I serve and consume the food.	

2. ◎ I wear my work clothes, but I know I need to change and stop using them while I serve and consume food. I intend to do this within six months.<br>3. ◎ I wear my work clothes, but I know I need to change and stop using them while I serve and consume food. I intend to do this within 30 days.<br>4. ◎ I do not wear my work clothes while I serve and consume food and I have changed my behavior for less than six months.<br>5. ◎ I do not wear my work clothes while I serve and consume food, and I have changed my behavior for more than six months.<br>6. ◎ My work environment does not require the use of work clothes.	
**7) Bringing work tools to the hospital restaurant.**<br>1. ◎ I take my work tools, and I do not feel like stopping taking my work tools to the restaurant.<br>2. ◎ I take my work tools, but I know I need to change and stop taking them to the restaurant. I intend to do this within six months.<br>3. ◎ I take my work tools, but I know I need to change and stop taking them to the restaurant. I intend to do this within 30 days.<br>4. ◎ I do not take my work tools to the restaurant and I changed my behavior for less than six months.<br>5. ◎ I do not take my work tools to the restaurant and I changed my behavior more than six months ago.<br>6. ◎ My work environment does not require the use of work tools.	
**8) Talking, sneezing, coughing while serving.**<br>1. ◎ I perform these actions, and I have no desire to stop doing these actions.<br>2. ◎ I perform these actions, but I know I need to change. I intend to do this within six months.<br>3. ◎ I perform these actions, but I know I need to change. I intend to do this within 30 days.<br>4. ◎ I have not performed these actions and I changed my behavior for less than six months.<br>5. ◎ I have not performed these actions and I changed my behavior for more than six months.	
**9) Eating/trying the food when serving.**<br>1. ◎ I eat while I am serving, and I do not feel like stopping eating while I'm serving.<br>2. ◎ I eat while I am serving, but I know I need to change, and I intend to do so in 6 months.<br>3. ◎ I eat while I am serving, but I know I need to change, and I intend to do so in 30 days.<br>4. ◎ I do not eat while serving and I changed my behavior less than six months ago.<br>5. ◎ I do not eat while serving and I changed my behavior more than six months ago.	
**10) Touching hair while serving.** Note that this includes wiggling your hair as well as scratching, ironing, or straightening your hair regardless of its length.<br>1. ◎ I perform this action, and I have no desire to stop doing this action.	

2. ◎ I perform this action, but I know I need to change. I intend to do this within six months.<br>3. ◎ I perform this action, but I know I need to change. I intend to do this within 30 days.<br>4. ◎ I did not do this and I changed my behavior less than six months ago.<br>5. ◎ I did not do this and I changed my behavior more than six months ago.	
**11) Setting the food on the plate with utensils used to serve.**<br>1. ◎ I arrange the food with the utensils used to serve me, and I do not feel like stopping setting the food on my plate with the utensils.<br>2. ◎ I arrange the food with the utensils used to serve me, but I know I need to change. I intend to do this within six months.<br>3. ◎ I arrange the food with the utensils used to serve me, but I know I need to change. I intend to do this within 30 days.<br>4. ◎ I do not arrange food with utensils used to serve me and I changed my behavior less than six months ago.<br>5. ◎ I do not arrange food with utensils used to serve me and I changed my behavior more than six months ago.	
**12) Removing food from the plate and returning it to the vats with your hands or available utensil.**<br>1. ◎ I return food and I do not feel like stopping returning the food to the vats.<br>2. ◎ I return food, but I know that I must change. I intend to do this within six months.<br>3. ◎ I return food, but I know that I must change. I intend to do this within 30 days.<br>4. ◎ I did not return food and I changed my behavior less than six months ago.<br>5. ◎ I did not return food and I changed my behavior more than six months ago.	
**13) Serving the food with your hands.** Note that this includes using your hands to pick up any food from the dispensing counter or if the food falls accidentally, you put it back on the plate using your hands. Example: salad, fried foods, seasonings, bread, etc.<br>1. ◎ I serve with my hands, and I do not feel like stopping serving with my hands.<br>2. ◎ I serve with my hands, but I know I need to change, and I intend to do it in 6 months.<br>3. ◎ I serve with my hands, but I know I need to change, and I intend to do so in 30 days.<br>4. ◎ I do not use my hands and I have changed my behavior for less than six months.<br>5. ◎ I do not use my hands and I have changed my behavior for more than six months.	
Thank you for your participation, your support was fundamental to the execution of this research!	
